# Mechanical stretch-induced osteogenic differentiation of human jaw bone marrow mesenchymal stem cells (hJBMMSCs) via inhibition of the NF-κB pathway

**DOI:** 10.1038/s41419-018-0279-5

**Published:** 2018-02-12

**Authors:** Xiaoyan Chen, Yuan Liu, Wanghui Ding, Jiejun Shi, Shenglai Li, Yali Liu, Mengjie Wu, Huiming Wang

**Affiliations:** 1 Department of Orthodontics, Affiliated Hospital of Stomatology, Medical College, Zhejiang University, Hangzhou, Zhejiang Province China; 2 Department of Liver Surgery, Ren Ji Hospital Affiliated to Shanghai Jiao Tong University School of Medicine, Shanghai, China; 3 Department of Oral Surgery, Affiliated Hospital of Stomatology, Medical College, Zhejiang University, Hangzhou, Zhejiang Province China; 4 Department of Orthodontics, Affiliated Hospital of Stomatology, Kunming Medical University, Kunming, Yunnan Province China; 5 Department of Oral Implantology, Affiliated Hospital of Stomatology, Medical College, Zhejiang University, Hangzhou, Zhejiang Province China

## Abstract

Severe malocclusion can contribute to several serious dental and physical conditions, such as digestive difficulties, periodontal disease, and severe tooth decay. Orthodontic treatment is mainly used to treat malocclusion. Forces in orthodontic tooth results in bone resorption on the pressure side and bone deposition on the tension side. Osteoblasts have been considered as the key component in bone regeneration on the tension side. However, the underlying mechanisms remain unclear. In this study, we focus on how mechanical stretch regulates the osteogenesis during orthodontic treatment. Human jaw bone marrow mesenchymal stem cells (hJBMMSCs) were isolated from healthy adult donors and cultured in regular medium (control) or osteogenic medium (OS). Under OS culture, hJBMMSCs presented osteogenic differentiation potentials, as evidenced by increased mineralization, enhanced calcium deposition, and upregulated expression of osteogenesis markers (ALP, osterix, and Runx). What’s more, the OS-induced osteogenesis of hJBMMSCs is associated with the dephosphorylation of IKK, activation of IKBα, and phosphorylation/nucleic accumulation of P65, which all indicated the inhibition of NF-κB activity. Overexpressing *P65* in hJBMMSCs, which could constantly activate NF-κB, prevented the osteogenic differentiation in the OS. After that, we applied the Flexcell tension system, which could cause mechanical stretch on cultured hJBMMSCs to mimic the tension forces during tooth movement. Mechanical stretch resulted in 3.5−fold increase of ALP activity and 2.4–fold increase of calcium deposition after 7 days and 21 days treatment, respectively. The expression levels of ALP, Run×2, and Osterix were also significantly upregulated. In the meantime, applying mechanical stretch on OS-cultured hJBMMSCs also dramatically promoted the OS-induced osteogenesis. Both OS and mechanical stretch downregulated NF-κB activity. By overexpressing *P65* in hJBMMSCs, neither OS nor mechanical stretch could induce their osteogenesis. These results indicated that, like OS induction, mechanical stretch-facilitated osteogenesis of hJBMMSCs by inhibiting NF-κB in the noninflammatory environments.

## Introduction

Orthodontic tooth movement is possible due to the remodeling ability of the surrounding bone and soft tissues. Orthodontic appliances are built to generate biomechanical force systems that produce the desired tooth and jaw movements needed to establish an ideal occlusion^1^. In orthodontic tooth movement, bone resorption occurs on the pressure side and bone deposition takes place on the tension side^2^. Osteoblasts have been considered as the key component in bone regeneration on the tension side^3^. They are derived from mesenchymal stem cells (MSCs) in the bone marrow and make up the organic bone matrix^4^. Osteoblasts and their precursor cells are sensitive to mechanical stimuli and have an ability to translate the mechanical signals into biological responses^5^. Various biological factors such as prostaglandin E2 (PGE), osteoprotegerin (OPG), bone morphogenetic proteins (BMPs), and nuclear factor-κB (NF-κB) have been established as important elements in osteogenic differentiation and bone remodeling^5,6^.

The transcription factor NF-κB represents a family of five mammalian proteins (c-rel, relA, p65, NF-κB1, and NF-κB2) that regulate a myriad of genes involved in immune and inflammatory responses, among many other functions^7^. NF-κB dimers reside in cytoplasm in an inactive state through interactions with κB inhibitors (IκBs). Upon activation, IκB kinase (IKK) phosphorylates IκB and facilitates its degradation. Dissociated NF-κB homodimers or hetertodimers then enter the nuclei and activate transcription^8,9^. Pro-inflammatory cytokines such as TNF and IL-17 can stimulate IKK-NF-κB and inhibit osteogenic differentiation of MSCs in inflammatory settings^8^. Activation of NF-κB blocks c-Jun N-terminal kinase (JNK) activity and other osteoblast proteins, such as MGP, Smad and Fra1, which eventually impedes osteogenic differentiation of MSCs^5,10,11^. However, with respect to orthodontic tooth movement in non-inflammatory environments, how mechanical signals affect NF-κB and regulate MSCs behavior remains unknown.

Like MSCs from other sites, such as the ilium and sternum, human jaw bone marrow MSCs (hJBMMSCs) have the potential to differentiate into osteoblasts^12,13^. Our previous study has revealed that inflammatory microenvironments impaired osteogenesis of human periodontal ligament tissue-derived MSCs by activating GSK-3β and NF-κB^4^. In the current study, we focus on how the mechanical stretch in non-inflammatory environments regulate the osteogenesis of hJBMMSCs. We isolated hJBMMSCs from healthy adult human donors and identified their stem cell properties. In vitro mechanical stretch and osteogenic medium (OS) both could facilitate osteogenesis of hJBMMSCs by inhibiting the NF-κB pathway. However, overexpression of *P65* constantly activated NF-κB and diminished the mechanical stretch or OS-induced osteogenic differentiation.

## Results

### Characterization of isolated hJBMMSCs

**Fig. 1:**
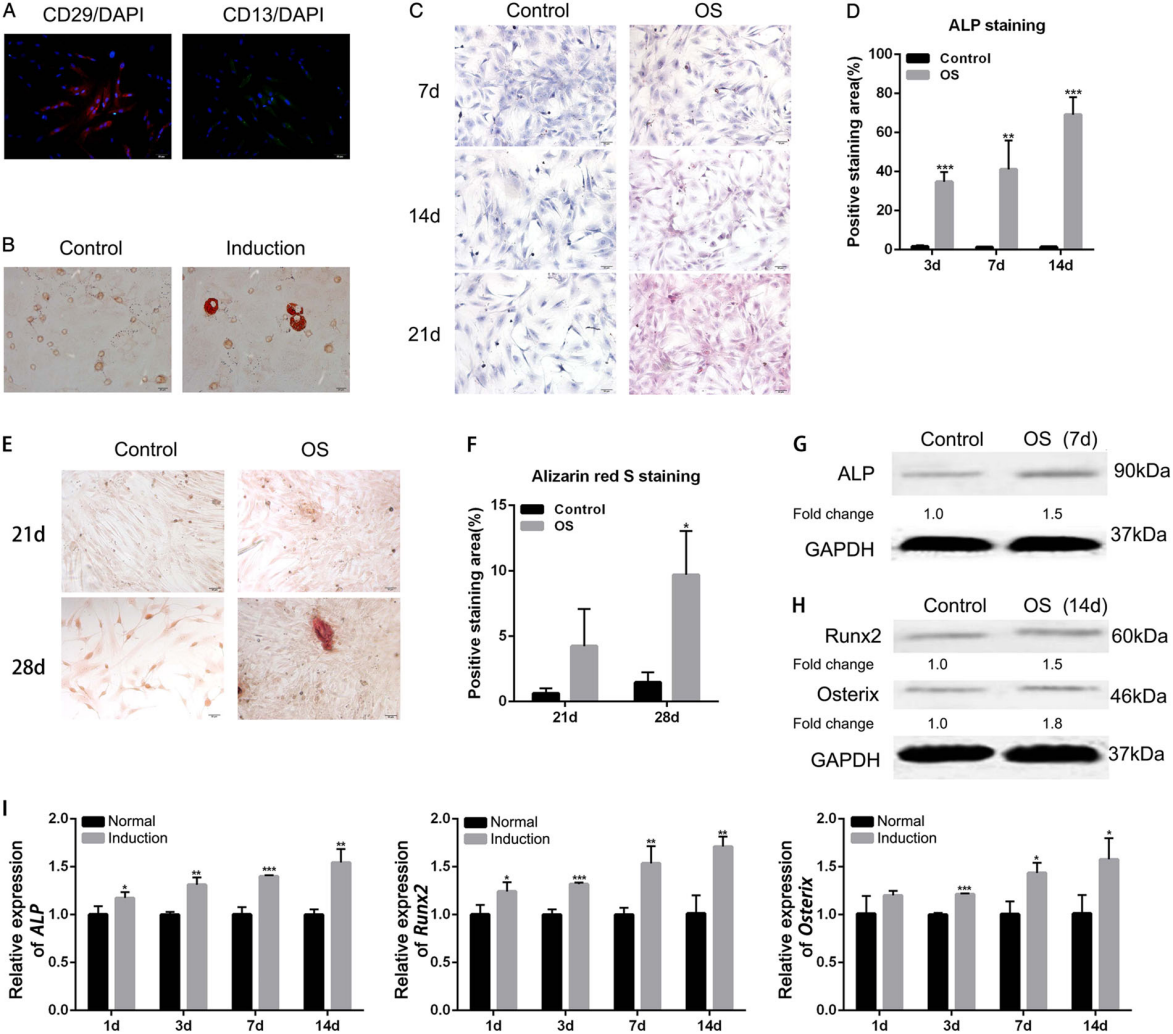
Isolated hJBMMSCs maintain stem cell properties and OS facilitates their osteogenic differentiation in vitro. **a** Immunofluorescence staining of CD29 and CD13 on hJBMMSCs. **b** Oil red staining indicated lipid restoration in hJBMMSCs after cultured in lipid-forming medium for 7 days. **c** Alkaline phosphatase (ALP) staining of hJBMMSCs cultured in regular medium (control) and osteogenic (OS) medium at day 7, 14, and 21. **d** Quantitative ALP activity in hJBMMSCs at day 7, 14, and 21. **e** Alizarin red staining of hJBMMSCs cultured in regular medium and OS at day 21 and 28. **f** Quantification of Alizarin red staining in hJBMMSCs. **g** Western blotting of ALP in hJBMMSCs cultured in regular medium and OS. **h** Western blotting of Runx2 and Osterix in hJBMMSCs cultured in regular medium and OS. **i** mRNA expression of *ALP, Runx2*, and *Osterix* in hJBMMSCs at day 1, 3, 7, and 14. (*n* = 6–10/group; *p < 0.05, **p < 0.01, ***p < 0.001)

Isolated hJBMMSCs were cultured in vitro and stained with stem cell-specific markers CD29 and CD13^14^. Immunofluorescence staining and flow cytometry analysis indicated high expression of CD29 and CD13 in >90% of the cultured hJBMMSCs, while low expression of CD45 and CD146 (Fig. 1a and Supplementary Fig. 1A). Lipid-forming medium treatment for 7 days in vitro resulted in lipid restoration in hJBMMSCs (Fig. 1b). Consistently, OS-cultured (osteogenic medium) hJBMMSCs indicated osteoblast characterization as evidenced by increased alkaline phosphatase activity (ALP, Fig. 1c, d) and intense alizarin red staining (Fig. 1e, f). ALP protein level increased 1.5-fold after 7 days incubation in OS (Fig. 1g). Osteoblast-specific transcription factors, Runx2 and Osterix, were also upregulated after OS induction (Fig. 1h, i). This result indicated that in vitro hJBMMSCs had stem cell properties and OS facilitated their osteogenic differentiation.

### Osteogenic differentiation of hJBMMSCs inhibited NF-κB activity

**Fig. 2:**
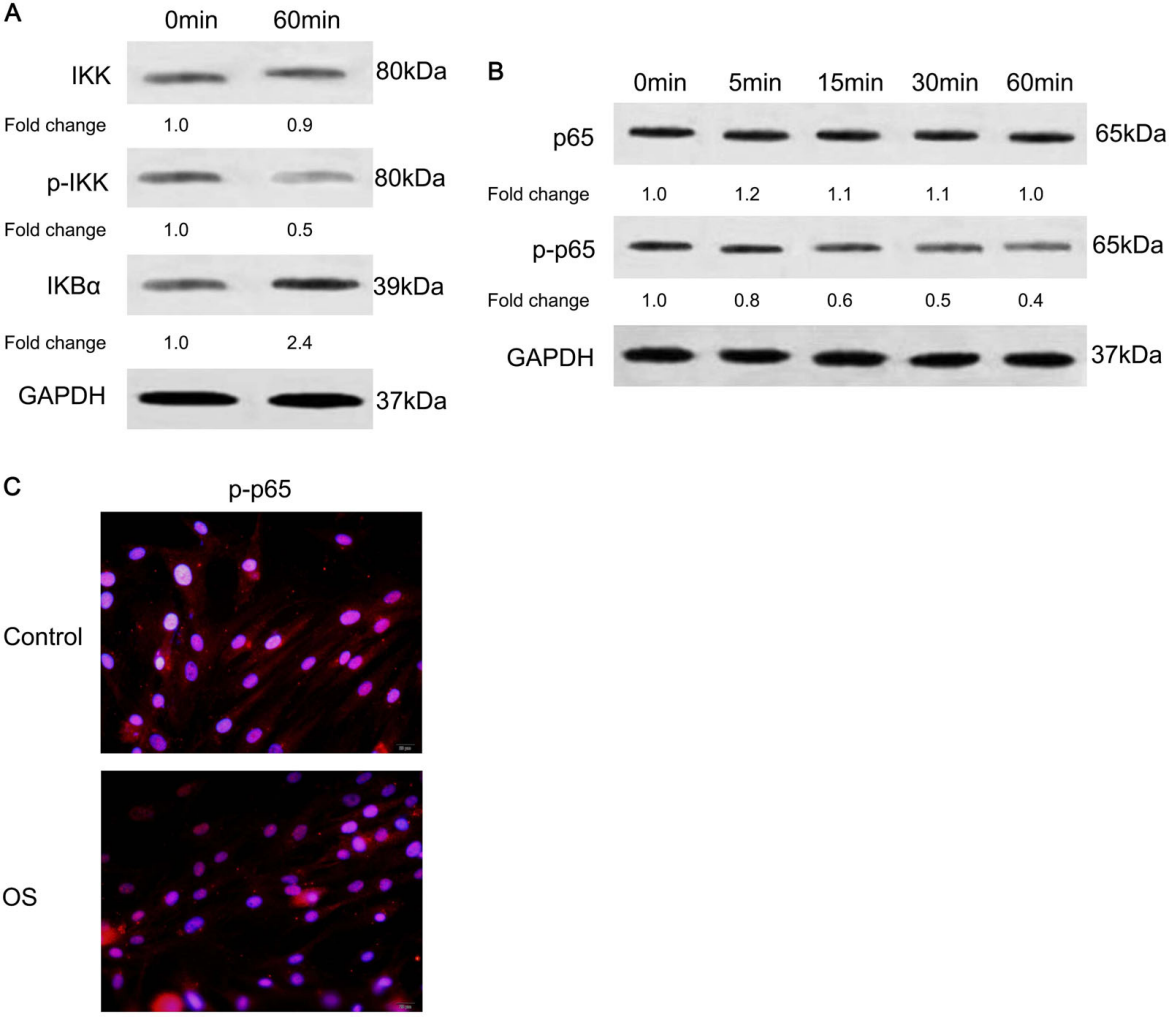
OS induces osteogenic differentiation of hJBMMSCs by inhibiting NF-κB activity. **a** Western blotting indicated decreased phosphorylation of IKK and increased IKBα in hJBMMSCs after 60 min induction by OS. **b** Phosphorylation of P65 decreased in hJBMMSCs in time-dependent manner after OS induction. **c** Immunofluorescence staining of phosphorylated P65 (p-P65) in hJBMMSCs. Less nucleic accumulation of p-P65 was demonstrated after OS induction

Next, we tested how NF-κB activity was regulated during the osteogenic differentiation of hJBMMSCs. Sixty minutes after OS induction, IKBα degradation was inhibited as p-IKK decreased and IKBα increased (Fig. 2a). Meanwhile, activity of P65 was downregulated after OS induction, as its phosphorylation and accumulation in nuclei were all inhibited (Fig. 2b, c and Supplementary Fig. 1B). This finding indicated that OS induction blocked the NF-κB activity of hJBMMSCs in a time-dependent manner.

### Activation of NF-κB blocked osteogenic differentiation

**Fig. 3:**
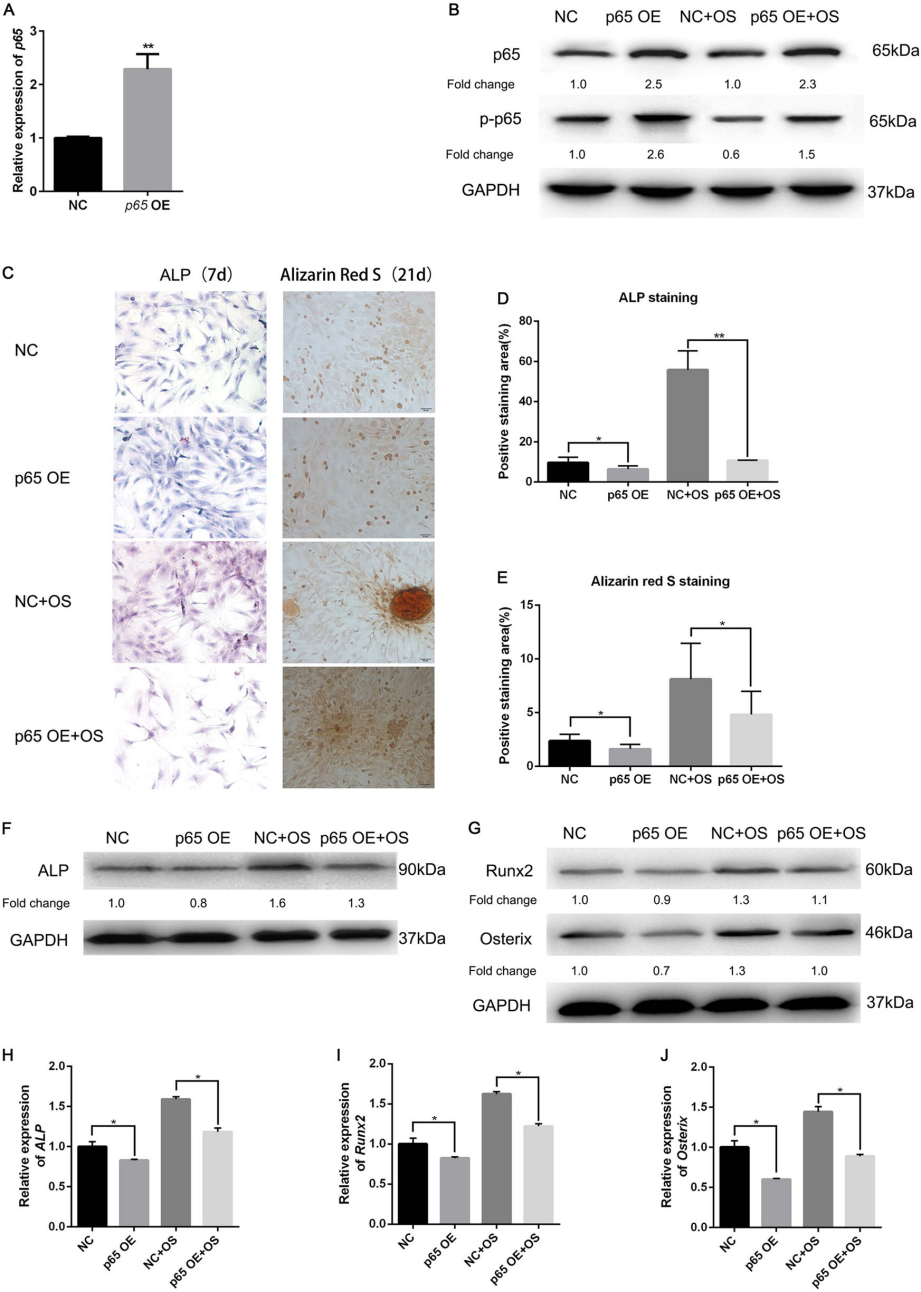
Constantly activating NF-κB blocks OS-induced osteogenic differentiation of hJBMMSCs. **a** mRNA expression of *P65* in hJBMMSCs that transfected with control vector (NC) or *P65* overexpression plasmid (p65 OE). **b** Protein level of P65 and p-P65 in hJBMMSCs transfected with control vector or p65 overexpression plasmid, cultured in regular medium or OS. **c** ALP staining (7 days) and Alizarin red staining (21 days) of hJBMMSCs after cultured in regular medium or OS. **d**–**e** Quantification of ALP staining and Alizarin red staining. **f**–**g** Western blotting of ALP, Runx2, and Osterix in hJBMMSCs. **h**–**j** mRNA expression of *ALP, Runx2*, and *Osterix* in hJBMMSCs (*n* = 6–10/group; *p < 0.05, **p < 0.01)

Since osteogenic differentiation inhibited NF-κB activity, we tried to ascertain how activated NF-κB affected the osteogenesis of hJBMMSCs. Plasmids consistently overexpressing *P65* were transfected into hJBMMSCs and the transfection efficiency was confirmed by quantitative reverse transcription PCR (qRT-PCR) and western blotting (Fig. 3a, b). Compared with the control group, hJBMMSCs overexpressing *P65* demonstrated reduced osteogenesis after OS induction, as evidenced by less ALP activity (Fig. 3c, d) and mineralization (Fig. 3d, e). ALP expression was diminished by overexpressed *P65*, as indicated by western blotting and qRT-PCR (Fig. 3f, h). Meanwhile, Runx2 and Osterix were also blocked in P65-pretreated hJBMMSCs (Fig. 3g, i, j). This result indicated that constant activation of NF-κB by overexpressing *P65* blocked osteogenic differentiation of hJBMMSCs.

### Mechanical stretch-facilitated osteogenesis of hJBMMSCs in vitro via diminishing NF-κB activity

**Fig. 4:**
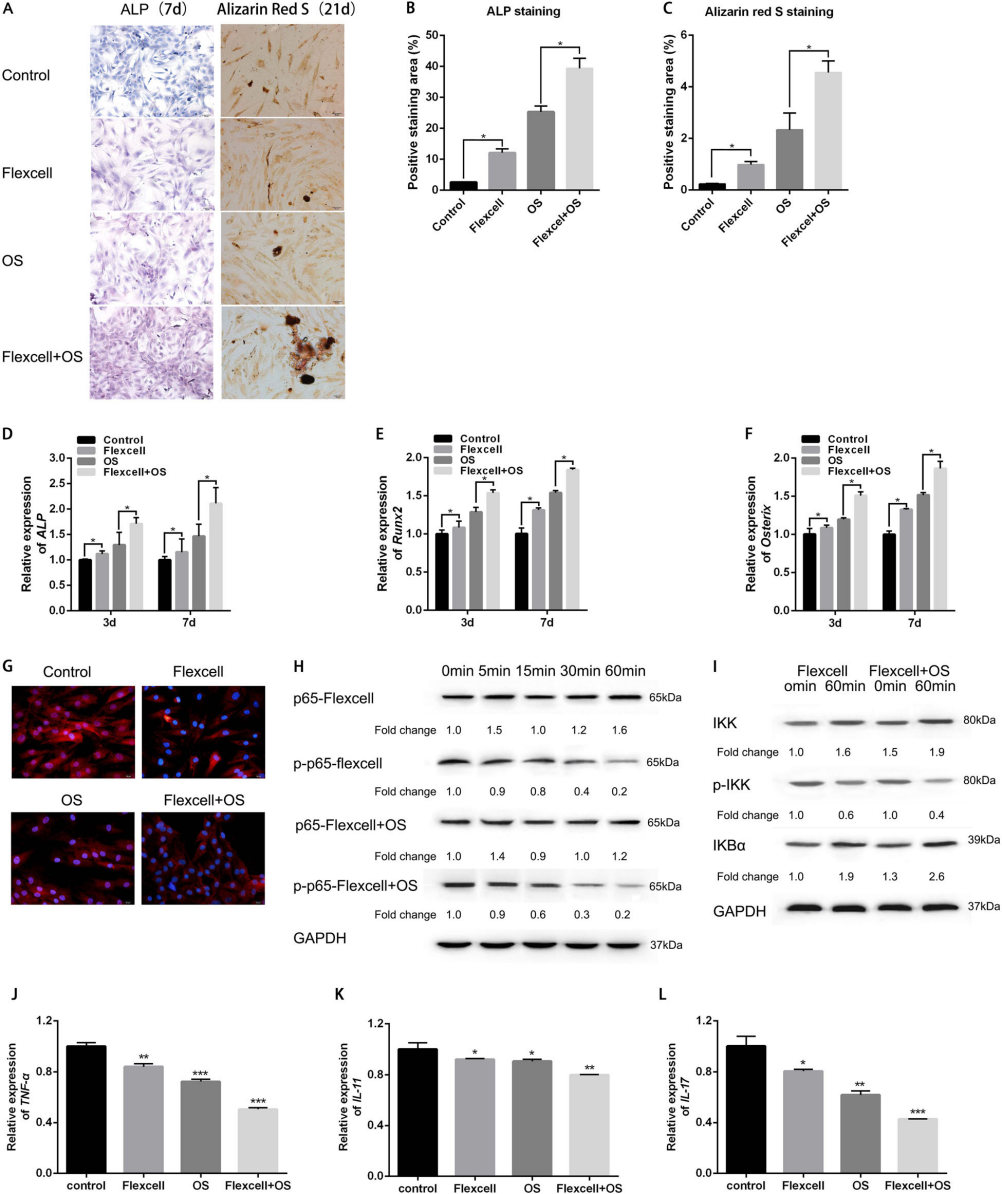
Inhibition of NF-κB is essential for the in vitro mechanical stretch-facilitated osteogenesis of hJBMMSCs. **a** ALP staining and Alizarin red staining of hJBMMSCs indicated that mechanical stretch-facilitated osteogenesis both in regular medium and OS. **b**–**c** Quantification of ALP staining and Alizarin red staining. **d**–**f** mRNA expression of *ALP, Runx2*, and *Osterix* in hJBMMSCs. **g** Immunofluorescence staining of p-P65 revealed that mechanical stretch reduced p-P65 expression level and its accumulation in the nuclei. **h**–**i** Mechanical stretch decreased p-P65 and p-IKK protein level, while increased IKBα level in a time-dependent manner. **j**–**l** mRNA expression of *TNF-α, IL-11*, and *IL-17.* (*n* = 6–10/group; *p < 0.05, **p < 0.01, ***p < 0.001)

The Flexcell tension system could induce mechanical stretch in vitro on cultured hJBMMSCs, which mimicked the scenario of tension stress during orthodontic tooth movement. We applied the Flexcell tension system on hJBMMSCs and found an osteogenic differentiation tendency, as indicated by massive positive staining of ALP (Fig. 4a, b) and mineralization (Fig. 4a, c). Meanwhile, Flexcell could facilitate the OS-induced osteogenesis of hJBMMSCs, which is accompanied with increased expression of Run×2 and Osterix (Fig. 4e, f, Supplementary Fig. 2A, B). Like how OS-induced osteogenesis could inhibit NF-κB activity, Flexcell-related osteogenic differentiation also eliminated P65 from nuclei (Fig. 4g and Supplementary Fig. 1C) and was positively correlated with downregulation of P65 in a time-dependent manner (Fig. 4h). IKBα degradation was inhibited by both Flexcell and OS (Fig. 4i). In addition, we also tested the expression of pro-inflammatory cytokines TNF-α, IL-17, and IL-11, which were regulated by NF-κB. qRT-PCR and ELISA revealed decreased expression and release of those cytokines after Flexcell and OS treatment (Fig. 4j–l, Supplementary Fig. 3). Thus, it can be inferred that mechanical stretch-induced osteogenesis via inhibition of NF-κB activity.

### Activation of NF-κB reversed mechanical stretch-mediated osteogenesis

**Fig. 5:**
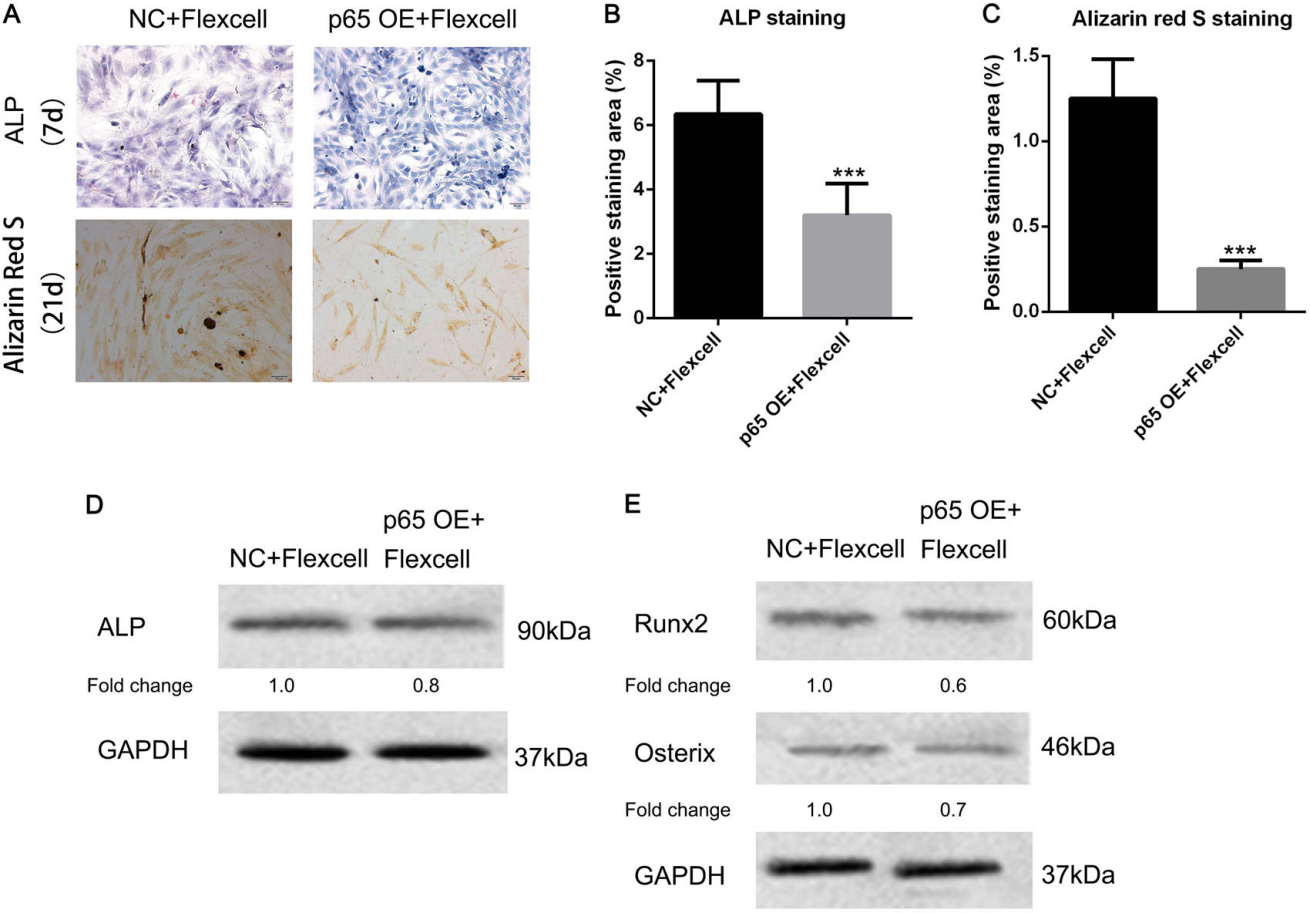
Constant activation of NF-κB reverses mechanical stretch-mediated osteogenesis of hJBMMSCs. **a** ALP staining and Alizarin red staining indicated that overexpression of *P65* in hJBMMSCs inhibited mechanical stretch-induced osteogenesis. **b–c** Quantification of ALP staining and Alizarin red staining. **d–e** Western blotting of ALP, Runx2, and Osterix in hJBMMSCs. (*n* = 6–10/group; ***p < 0.001)

To further study the role of NF-κB in mechanical stretch-induced osteogenesis, we constantly overexpressed *P65* in hJBMMSCs and employed the Flexcell tension system. After Flexcell and OS treatment, lighter staining of ALP and reduced mineralization were observed in P65 overexpressed hJBMMSCs, when compared with the control group (Fig. 5a–c). Intracellular ALP level decreased 25% by over-activated P65 in the setting of Flexcell treatment (Fig. 5d). Osteoblast-related transcription factors Run×2 and Osterix were also inhibited by activated P65 (Fig. 5e). Therefore, mechanical stretch-induced osteogenic differentiation of hJBMMSCs was diminished by unlimited activation of NF-κB.

## Discussion

Orthodontic tooth movement is a result of bone remodeling by differentially generating osteoblasts and osteoclasts in the pressure and tension areas around teeth. However, how mechanical stress during orthodontic tooth movement regulates osteoblast differentiation is still unknown. In this study, we isolated hJBMMSCs from healthy adult human donors and confirmed their potential for osteogenesis in osteogenic medium. The osteogenic differentiation of hJBMMSCs in vitro was achieved by inhibiting NF-κB activity. Meanwhile, using the Flexcell tension system to apply mechanical stretch on hJBMMSCs also facilitated their osteogenesis. NF-κB activity was blocked in the mechanical stretch-induced osteogenic differentiation, while constant activation of NF-κB diminished the differentiation. Our study is the first to confirm the osteogenic role of hJBMMSCs during mechanical stretch, which is dependent on inhibition of NF-κB.

hJBMMSCs are postnatal multipotent stem cells residing in the maxilla and mandible^13^. The osteoclasts and osteoblasts that present during orthodontic tooth movement are derived from hJBMMSCs^15,16^. Compared with bone marrow MSCs from appendicular bones, hJBMMSCs have been shown to have distinct characteristics in terms of differentiation traits^12^. hJBMMSCs have an increased proliferation rate and bone-forming capacity in vitro and in vivo, but less chondrogenic and adipogenic potential compared with BMMSCs from long bones^16^. Unlike craniofacial MSCs, which are widely studied in plasticity and regeneration^17^, the differentiation of hJBMMSCs has been mainly discussed under inflammatory environments, such as periodontitis, wounds, and abscesses^18,19^. Inflammation usually impairs multipotency of hJBMMSCs and results in weakened bone regeneration^4^. Nevertheless, how non-inflammatory stress, like mechanical stress, affects the behaviors of hJBMMSCs is poorly understood. In this study, we isolated hJBMMSCs from healthy humans and cultured them in vitro. Osteogenic medium and mechanical stretch both induced their osteogenic differentiation. After a mechanical stretch induction, osteoblast-specific transcription factors expression and mineralization in hJBMMSCs were increased. Since tension resulting from orthodontic forces facilitates bone regeneration, our results indicated that hJBMMSCs could be the source for osteogenesis after a mechanical stretch induction.

The Flexcell tension system was employed in our study to mimic the mechanical tension during orthodontic tooth movement^20^. Mechanical loading is one of the most important physical stimuli for osteoblast differentiation, for both new bone generation and maintenance^21,22^. Mechanical stretch on MSCs regulates Run×2 activation and favors osteoblast differentiation through the activation of the MAPK signal transduction pathways and Ras/Raf-dependent ERK1/2 activation^23,24^. In mature osteoblasts, mechanical stimuli are transferred into biological responses by Ca^2+^ accumulation and MAPK pathway activation, such as ERK1/2 and JNK activation, which maintains the property of osteoblasts^25,26^. In our study, Flexcell-induced mechanical stretch-facilitated osteogenesis of hJBMMSCs by upregulation of Runx2 and Osterix, and promoted the ALP accumulation and mineralization. Furthermore, mechanical stretch-induced osteogenesis was time-dependent. These results confirmed that bone regeneration on the tension side of orthodontic tooth movement could result from the osteogenesis of hJBMMSCs.

NF-κB is considered to inhibit the osteogenic pathway in MSCs both in vivo and in vitro^4,10^. NF-κB inhibits osteogenesis of MSCs by promoting β-catenin degradation in inflammatory microenvironments^10^. Even though some earlier studies showed that NF-κB activation by RANKL or mechanical loading promoted osteogenic differentiation of MSCs^6,27^, our results revealed that both osteogenic differentiation medium and mechanical stretch blocked NF-κB activity. Deactivation of P65, accumulation of IKBα, and decreases of phosphorylated IKK in hJBMMSCs were observed during the induced osteogenesis. Meanwhile, constantly activated NF-κB via overexpression of *P65* in hJBMMSCs efficiently blocked the OS and mechanical stretch-mediated osteogenic differentiation. Our results revealed that the mechanical stretch in non-inflammatory environments facilitates osteogenesis by inhibiting NF-κB activity.

In summary, our study suggested that the mechanical stretch in non-inflammatory environments facilitated osteogenesis of hJBMMSCs by blocking NF-κB activity. Constantly activating NF-κB could diminish osteoblast generation from hJBMMSCs and delay new bone generation. Our study revealed the critical role of NF-κB in regulation of bone generation during orthodontic tooth movement. This finding could lead to novel orthodontic appliances and improved jaw-related plasticity.

## Methods

### Isolation of hJBMMSCs and culture

hJBMMSCs were obtained from healthy adult human donors (aged 18–35 years) undergoing orthognathic surgery. Jaw bone debris was rinsed with α-minimum essential medium (α-MEM, Gibco BRL, MD, USA) containing 100 U/mL of penicillin and 100 mg/mL of streptomycin (Invitrogen, Carlsbad, CA, USA) three times. The debris was cut into 1 mm^3^ pieces and passed through a 70-mm strainer to obtain single-cell suspensions. Both the cell suspensions and jaw bone pieces were seeded into 10-cm dishes and cultured with α-MEM supplemented with 15 % fetal bovine serum (FBS, Gibco BRL) and incubated in 5% CO_2_ at 37 °C. hJBMMSCs at passage three were used for the following experiments. NF-κB P65 plasmids (Santa Cruz, CA, USA) were transfected into hJBMMSCs to overexpress *P65*.

### Induction of osteogenic differentiation

A total of 2 × 10^5^ cells were plated onto 6−well plate and cultured with α-MEM. When cell confluence reached 80%, the culture medium was switched to osteogenic medium (α-MEM containing 100 nM dexamethasone, 50 mg/mL ascorbic acid, and 5 mM β-glycerophosphate (Sigma, MO, USA)) and cultured for an additional 7 to 21 days. Calcium nodule deposit was detected by 2% Alizarin red (pH 4.2, Sigma). Ten percent cetylpyridinium chloride was used as Alizarin red quantification and absorbance was measured at 562 nm.

### Immunofluorescence

Cultured hJBMMSCs were stained by immunofluorescence after treatment. Primary phospho-P65 mAb (ab86299, Abcam, Cambridge, United Kingdom), CD29 mAb (303005, Biolegend, San Diego, CA) or CD13 mAb (ab7417, Abcam) were used and followed by donkey anti-rabbit FITC/CY3 conjugated secondary mAb (Life Technologies, Grand Island, NY). Cells were treated with DAPI (Vector Labs), and evaluated blindly by counting labeled cells in ten HPFs (high-power fields).

### Flow cytometry

Cultured hJBMMSCs were isolated and stained with primary CD29 mAb (303005, Biolegend), CD13 mAb (ab7417, Abcam), CD45 mAb (D9M8I, Cell Signaling Technology, Beverly, MA), and CD146 mAb (ab75769, Abcam), which was followed by donkey anti-rabbit FITC/CY3 conjugated secondary mAb (Life Technologies). hJBMMSCs were then analyzed on a FACSCalibur cytometer (BD Biosciences) and identified as CD29^+^CD13^+^CD45^−^CD146^−^.

### Employment of Flexcell system for in vitro mechanical stretch

hJBMMSCs were plated at 5 × 10^5^ cells/well in a Flexcell biaxial 6-well plate overnight. The medium was replaced by fresh basal medium or OS 24 h before cells being subjected to sustained mechanical stretch (12% at 0.5 Hz) by the Flexcell FX-4000 tension system (Flexcell International Corp., USA). Cell layers were then collected for further analysis.

### Total RNA extraction and qRT-PCR

Total RNA was extracted from in vitro culture samples using TRIzol reagent (Invitrogen, Grand Island, NY, USA). Reverse transcriptase-polymerase chain reaction was performed with 1 mg of RNA using the PrimeScript RT reagent kit (TaKaRa, Dalian, China). Related genes were quantified by real-time PCR using the SYBR Premix Ex Taq II kit (TaKaRa) and the CFX96 Touch Real-Time PCR Detection System (Bio-rad, Hercules, CA, USA).

### Protein isolation and western blotting analysis

Total proteins were extracted with lysis buffer (10 mM Tris-HCL, 1 mM EDTA, 1% sodium dodecyl sulfate, 1% Nonidet P-40, 1:100 proteinase inhibitor cocktail, 50 mM b-glycerophosphate, and 50 mM sodium fluoride). Aliquots of 20–60 mg per sample were separated by 10% sodium dodecyl sulfate-polyacrylamide gel electrophoresis, transferred to the polyvinylidene fluoride membranes and blocked with 5% nonfat milk powder in PBST (PBS with 0.1% Tween). Next, they were incubated with the following primary antibodies overnight: anti-Osterix, anti-Runx2, anti-ALP, anti-P65, anti-p-P65, anti-IKBα, anti-IKK, anti-p-IKK (Cell Signaling Technology). The membranes were then incubated with horseradish peroxidase-conjugated anti-mouse or anti-rabbit IgG secondary antibodies (Boster, Wuhan, China). The blots were visualized using an enhanced chemiluminescence kit (Amersham Biosciences, Piscataway, NJ, USA) according to the manufacturer’s recommended instructions.

### Statistical analysis

All results are presented as mean ± S.D. from at least three independent experiments and were analyzed by a two-tailed unpaired Student’s *t*-test using the SPSS software (IBM, Armonk, NY, USA). *p*-value < 0.05 was considered as statistically significant.

## Electronic supplementary material

Supplementary caption

Supplementary Fig. 1

Supplementary Fig. 2

Supplementary Fig. 3
