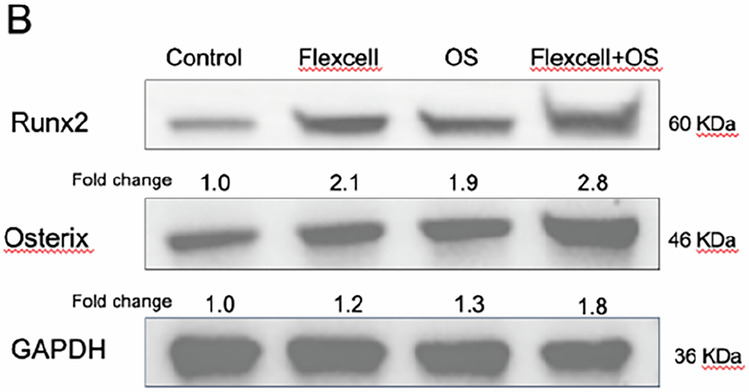# Correction: Mechanical stretch-induced osteogenic differentiation of human jaw bone marrow mesenchymal stem cells (hJBMMSCs) via inhibition of the NF-κB pathway

**DOI:** 10.1038/s41419-025-07763-1

**Published:** 2025-07-22

**Authors:** Xiaoyan Chen, Yuan Liu, Wanghui Ding, Jiejun Shi, Shenglai Li, Yali Liu, Mengjie Wu, Huiming Wang

**Affiliations:** 1 Department of Orthodontics, Affiliated Hospital of Stomatology, Medical College, Zhejiang University, Hangzhou, Zhejiang Province China; 2 Department of Liver Surgery, Ren Ji Hospital Affiliated to Shanghai Jiao Tong University School of Medicine, Shanghai, China; 3 Department of Oral Surgery, Affiliated Hospital of Stomatology, Medical College, Zhejiang University, Hangzhou, Zhejiang Province China; 4 Department of Orthodontics, Affiliated Hospital of Stomatology, Kunming Medical University, Kunming, Yunnan Province China; 5 Department of Oral Implantology, Affiliated Hospital of Stomatology, Medical College, Zhejiang University, Hangzhou, Zhejiang Province China

Correction to: *Cell Death & Disease* 10.1038/s41419-018-0279-5, published online 12 February 2018

In the originally published Figure 5a, the Alizarin Red S (21d) staining images of NC+ Flexcell-treated cells were inadvertently duplicated. This error can be partly attributed to the similarities among the images. The correct Alizarin Red S (21d) images for NC+ Flexcell-treated cells have been included.

Originally published
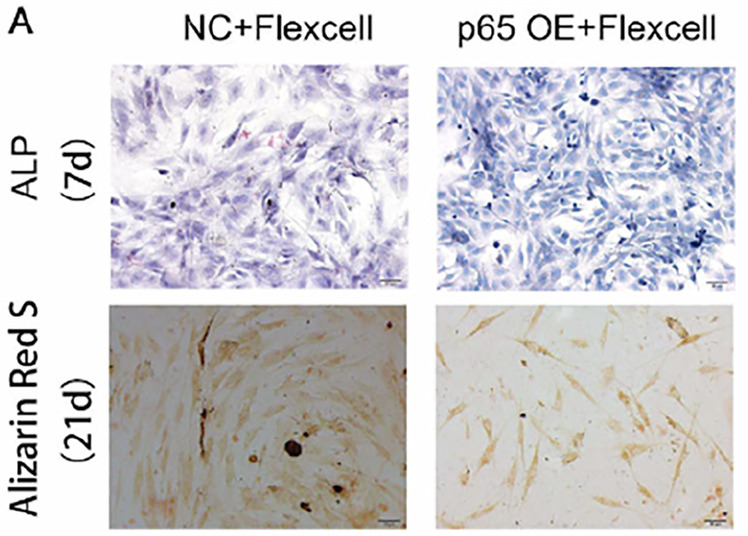


Corrected version
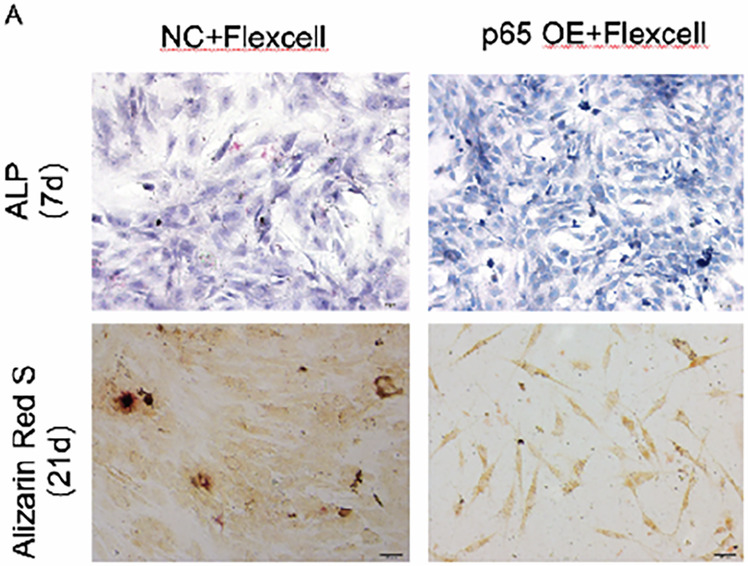


In the originally published Supplementary Figure 2b, the Runx2 protein band closely resembled that of Osterix. To validate the trends of these two proteins, we repeated the relevant experiments, and the results confirmed that the expression patterns of both proteins aligned with our previous findings. To prevent any confusion, we have updated the relevant experimental figures.

Originally published
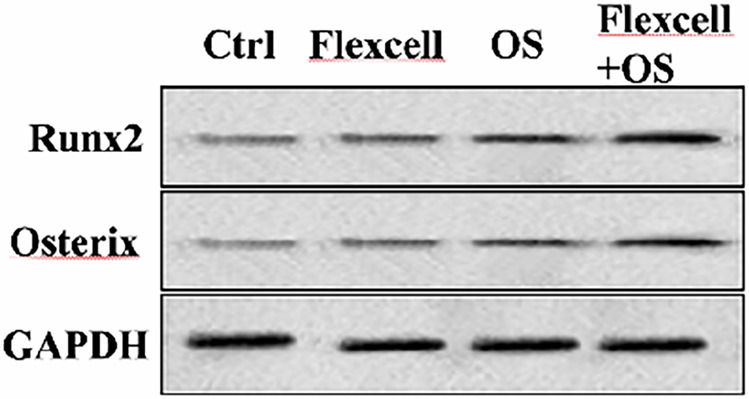


Corrected version