# Supplementary material for: Mechanical stretch-induced osteogenic differentiation of human jaw bone marrow mesenchymal stem cells (hJBMMSCs) via inhibition of the NF-κB pathway
Source: Cell Death Dis. 2018 Feb 12;9(2):207. doi: 10.1038/s41419-018-0279-5 (PMC5833399; doi:10.1038/s41419-018-0279-5)
Supplement: Supplementary file 1 — Supplementary caption [file 41419_2018_279_MOESM1_ESM.docx]

**Supplementary Fig. 1**

(A) Flow cytometry analysis of CD29, CD13, CD45 and CD146 on hJBMMSCs; (B) Quantification of phospho-P65 (p-p65) fluorescence density in hJBMMSCs after OS treatment. (C) Quantification of p-p65 fluorescence density in hJBMMSCs after OS or Flexcell treatment. (n=6-10/group; ***p<0.001)

**Supplementary Fig. 2**

(A-B) hJBMMSCs were cultured in regular medium or OS, and treated by Flexcell tension system or control (no tension applied) spontaneously. Western blotting indicated expression changes of ALP, Runx2 and Osterix.

**Supplementary Fig. 3**

(A-B) Quantification of hJBMMSCs released TNF-α and IL-17 level by ELISA. (n=6-10/group; *p<0.05, **p<0.01, ***p<0.001)
