# Supplementary figures and images for: Mechanical stretch-induced osteogenic differentiation of human jaw bone marrow mesenchymal stem cells (hJBMMSCs) via inhibition of the NF-κB pathway
Source: Cell Death Dis. 2018 Feb 12;9(2):207. doi: 10.1038/s41419-018-0279-5 (PMC5833399; doi:10.1038/s41419-018-0279-5)

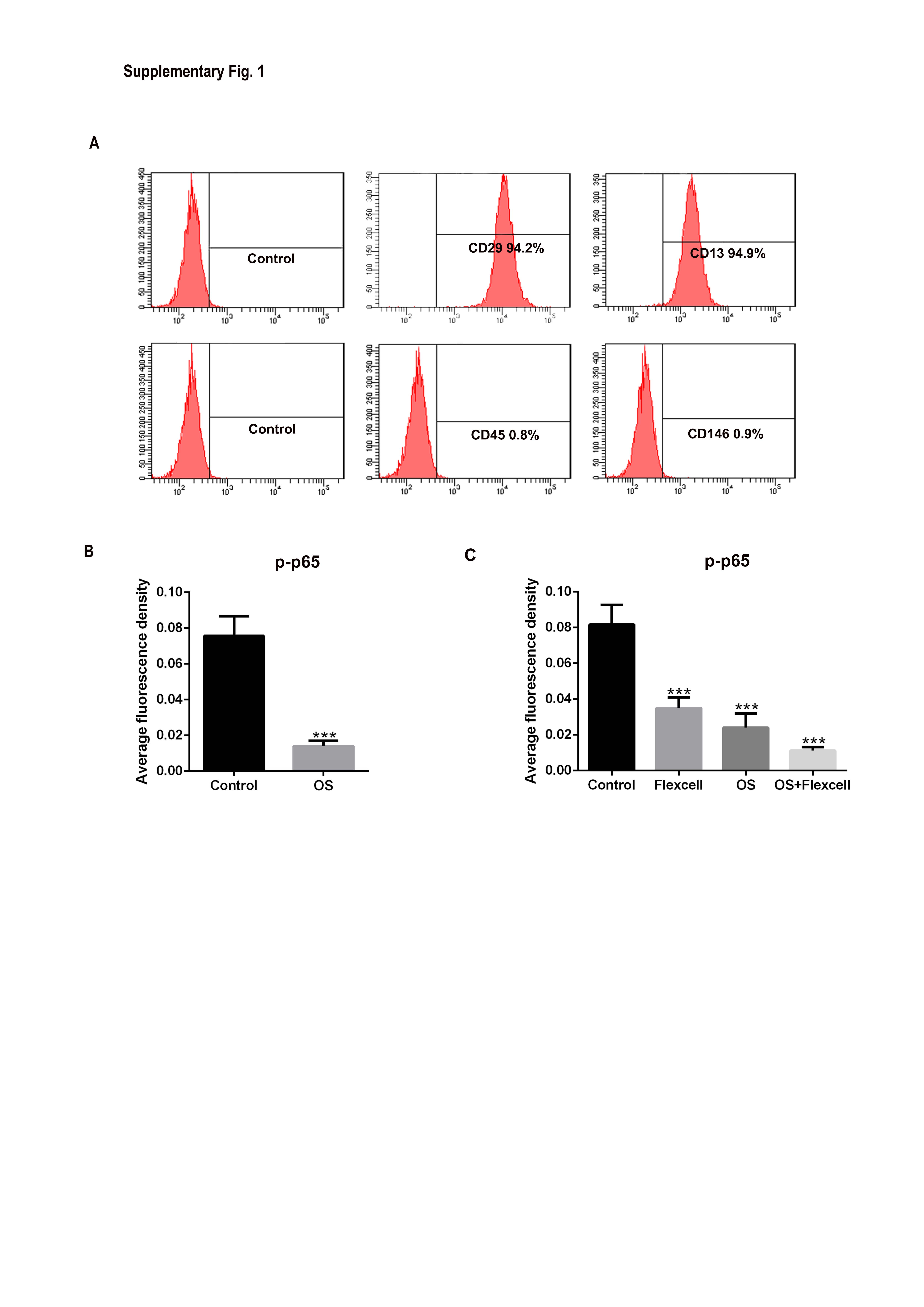

Supplement: Supplementary file 2 — Supplementary Fig. 1 [file 41419_2018_279_MOESM2_ESM.jpg]

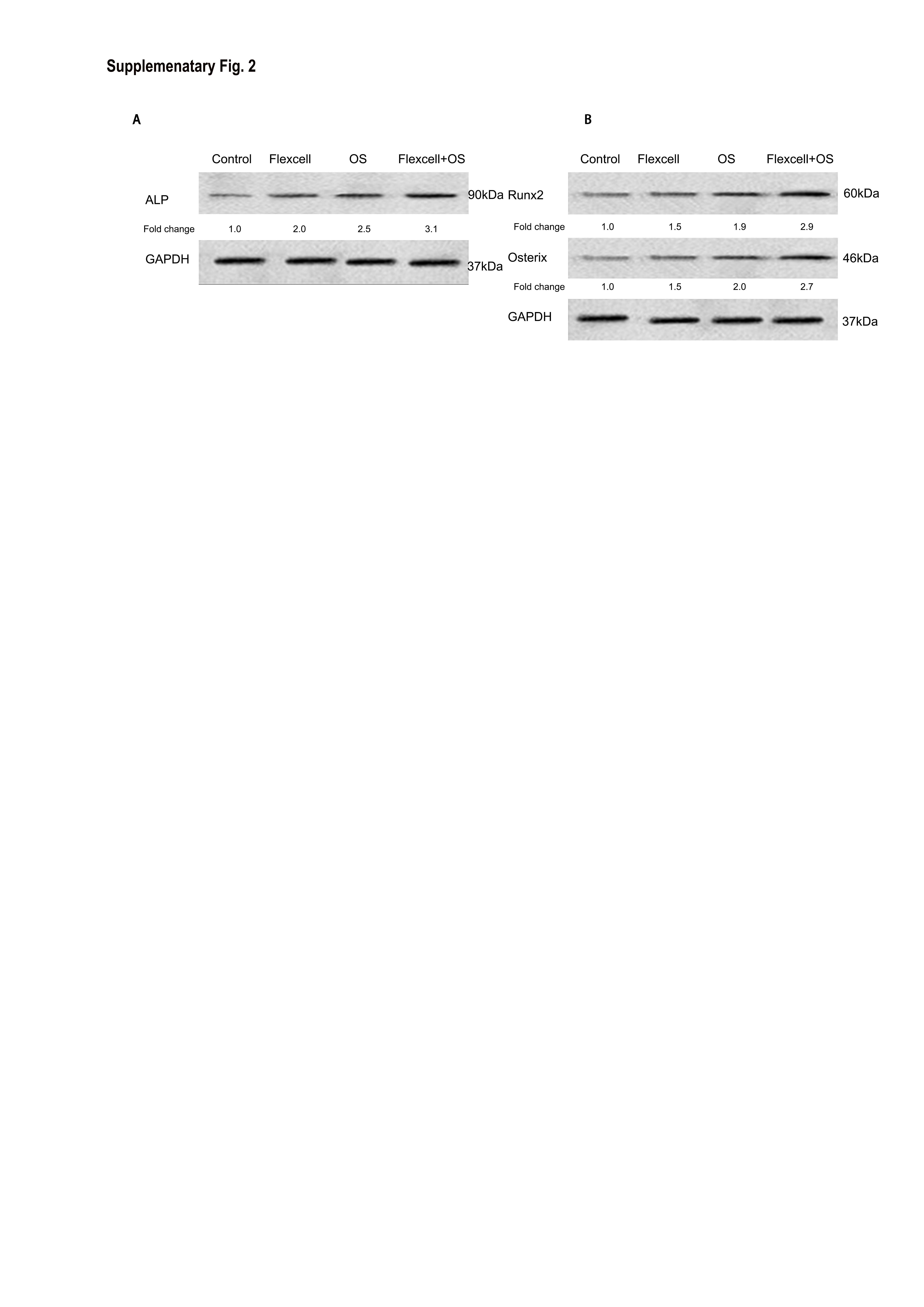

Supplement: Supplementary file 3 — Supplementary Fig. 2 [file 41419_2018_279_MOESM3_ESM.jpg]

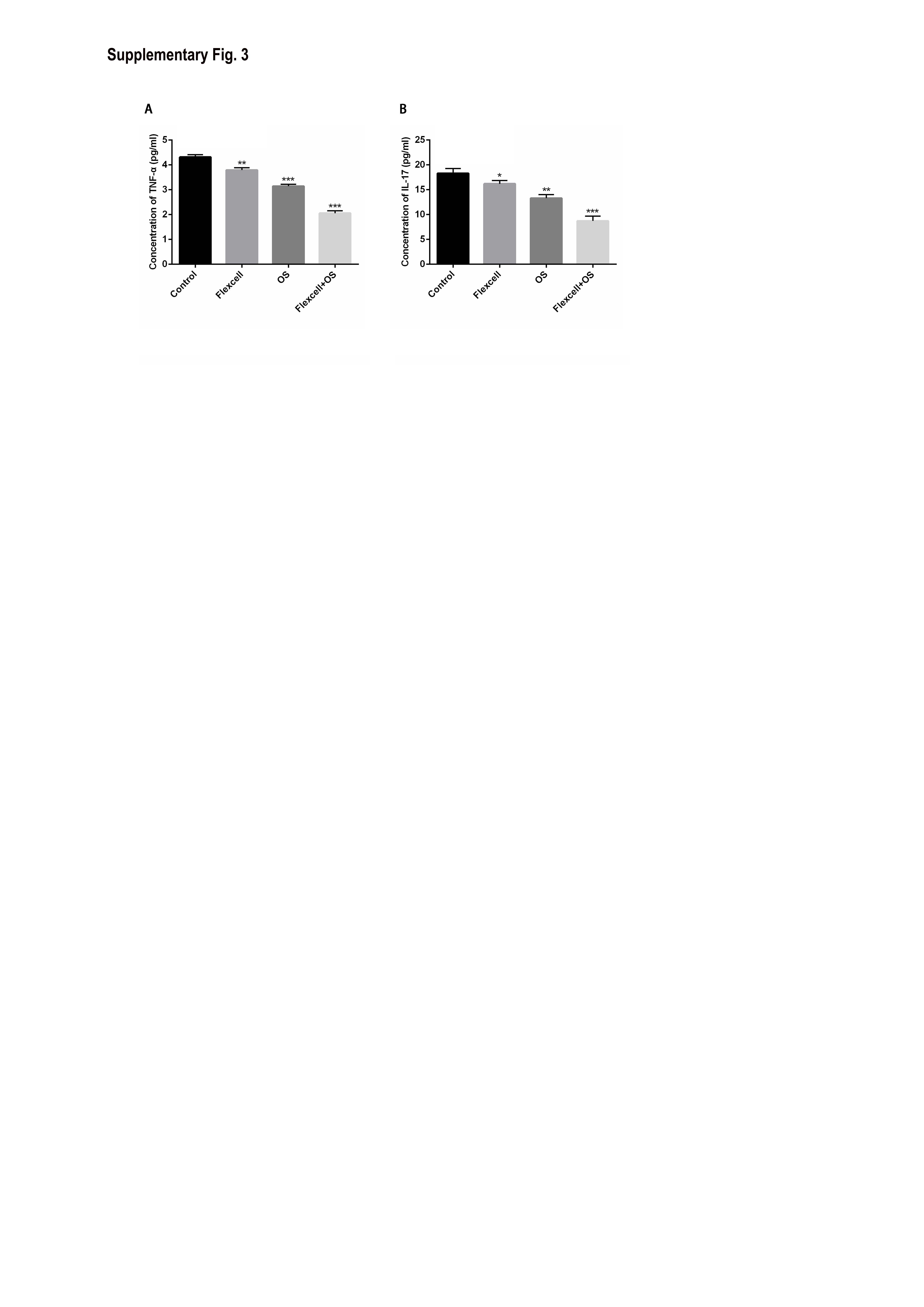

Supplement: Supplementary file 4 — Supplementary Fig. 3 [file 41419_2018_279_MOESM4_ESM.jpg]
